# The Effect of Haemodialysis Access Types on Cardiac Performance and Morbidities in Patients with Symptomatic Heart Disease

**DOI:** 10.1371/journal.pone.0148278

**Published:** 2016-02-05

**Authors:** Min-Kai Chuang, Chin-Hao Chang, Chih-Yang Chan

**Affiliations:** 1 Department of Surgery, National Taiwan University Hospital Yun-Lin Branch, Yun-Lin, Taiwan; 2 Department of Medical Research, National Taiwan University Hospital, Taipei, Taiwan; 3 Department of Surgery, National Taiwan University Hospital, Taipei, Taiwan; Hospital Universitario LA FE, SPAIN

## Abstract

**Background:**

Little is known about whether the arteriovenous type haemodialysis access affects cardiac function and whether it is still advantageous to the uremic patient with symptomatic heart disease.

**Methods:**

We conducted a retrospective comparative study. Patients with heart disease and end-stage renal disease that had a new chronic access created between January 2007 and December 2008 and met the inclusion criteria were assessed. The endpoint was major adverse event (MAE)-free survivals of arteriovenous access (AVA) and tunneled cuffed double-lumen central venous catheter (CVC) groups. Whether accesses worsened heart failure was also evaluated.

**Results:**

There were 43 CVC patients and 60 AVA patients. The median follow-up time from access creation was 27.6 months (IQR 34.7, 10.9~45.6). Although CVC patients were older than AVA patients (median age 78.0, IQR 14.0 vs. 67.5, IQR 16.0, respectively, p = .009), they manifested non-inferior MAE-free survival (mean 17.1, 95% CI 10.3~24.0 vs. 12.9, 95% CI 8.5~17.4 months in CVC and AVA patients, respectively, p = .290). During follow-up, more patients in the AVA group than in the CVC group deteriorated in heart failure status (35 of 57 vs. 10 of 42, respectively, odds ratio 5.1, p < .001). Preoperative-postoperative pairwise comparison of echocardiographic scans revealed an increased number of abnormal findings in the AVA group (Z = 3.91, p < .001), but not in the CVC group.

**Conclusions:**

In patients with both symptomatic heart disease and end stage renal disease (ESRD), CVC patients showed non-inferior MAE-free survival in comparison to those in the AVA group. AV type access could deteriorate heart failure. Accordingly, uremic patients with symptomatic heart disease are not ideal candidates for AV type access creation.

## Introduction

An arteriovenous fistula (AVF) is the preferred method of haemodialysis access as it provides the safest, and most reliable and durable route.[1–3] Conversely, patients dialyzing with a tunneled cuffed double-lumen central venous catheter (CVC) tend to experience an inferior outcome.[4,5] Despite this evidence, many patients begin and chronically maintain regular haemodialysis via the CVC.[4–6] Nevertheless, the creation of an AVF is not without limitations, including variation in clinical evaluation of what constitutes “good veins” and a patient’s suitability for an AVF, [2] the timing of placement, [7] rate of failure-to-mature, variation in clinical decision regarding the placement of a prosthetic arteriovenous graft (AVG), and the potentially negative effect on the heart.[8,9]

Patients with chronic kidney disease (CKD) could present cardiac dysfunction. Recent studies have revealed that advanced CKD at baseline is associated with progressive worsening in cardiac structure and function.[10,11] Furthermore, the left-to-right shunting of blood through an AVF can significantly increase the preload on the heart [12] and presents as a major concern when dialyzing a uremic patient with heart disease and symptomatic congestive heart failure (CHF). Studies have shown that an AVF decreases systemic vascular resistance, causing increased stroke volume and cardiac output in order to maintain blood pressure, leading to left ventricular volume overload.[13]

The impact of AVF on cardiac function and clinical outcome has yet to be thoroughly elucidated, [14,15] resulting in the propagation of controversy regarding the technique and inconsistent selection of access types for patients with heart failure. Due to a lack of definitive criteria by which to stratify patients for different access routes, more research is necessary to identify patients whose heart failure might deem them intolerant to additional flow and volume overload from an AVF.

We hypothesized that an AVA could impact on cardiac function when patient had an existing heart disease. Accordingly, we aimed to identify factors predicting worsening in heart failure symptoms due to the increasing volume load from an arteriovenous access (AVA), thereby identifying patients who would lose benefit from a functioning AVA. The first objective of this study was to evaluate the clinical outcome, cardiac function change, and adverse events of patients with an AVA and symptomatic heart disease. The second objective was to compare echocardiographic parameters and clinical outcomes between patients with different types of primary vascular access for haemodialysis.

## Materials and Methods

The Institutional Research Ethics Committee of National Taiwan University Hospital approved this study. The procedures, including obtaining informed consent, were conducted in accordance with the ethical standards of the *Declaration of Helsinki*. Of note, specifically informed consent to participate in this study could not be obtained because of retrospective study design. Patient records/information was anonymized and de-identified prior to analysis.

### Patients

This study included all patients presenting with both heart disease and end-stage renal failure who had a new chronic access created between January 2007 and December 2008 at the Department of Surgery of National Taiwan University Hospital. Of note, each physician/surgeon’s clinical and ultrasonic evaluation of the patient’s suitability for an AVF/AVG or CVC varied and was left to his or her own discretion. Patients excluded from the study included those who had a CVC as a temporary access before receiving another type of maintaining dialysis access, as well as those who switched to peritoneal dialysis, had kidney transplantation, or changed to another type of haemodialysis vascular access during follow-up (certain CVC patients who had experienced recurrent catheter-related infections were proposed an AVA and therefore excluded from this study).

Patient baseline demographic information, including age, gender, body size, and comorbidities were collected in a database. Six comorbidities (excluding heart status and renal disease, which were present in all patients) were defined, but not graded, according to the reporting standards of the Society for Vascular Surgery as follows: (1) diabetes (history of using hypoglycemic agents or insulin, or a diagnosis of diabetes); (2) hypertension (history of using antihypertensive agents) (3) hyperlipidemia (history of dietary or pharmacologic intervention); (4) tobacco use (current smoker, or history of smoking in the preceding 10 years); (5) carotid or cerebral vascular disease (evidence of disease determined by duplex scan or other accepted noninvasive test or arteriogram, transient or temporary stroke, or complete stroke with permanent neurologic deficit or acute stroke); (6) pulmonary status (chronic parenchymal x-ray changes, impaired pulmonary function tests, medical use of supplemental oxygen, pulmonary hypertension, or documented chronic lung disease). Two additional morbidities that may affect clinical outcome were also recorded: peripheral artery disease (PAD; history of lower extremity revascularization, digit or extremity amputation, claudication and ischemic extremity changes, or gangrene); and dependence in activities of daily living (ADL; based on an evaluation of the functional dependence of patients in bathing, dressing, using a toilet, transferring, continence, and feeding).[16]

### Definitions of heart diseases

Although cardiac disease was present in all patients, the condition varied in entity and severity was evaluated in greater detail. First, a diagnosis of CHF, atrial fibrillation, or coronary artery disease (CAD) was sought. CHF was defined by a documented clinical functional classification, class 2 or above, according to the criteria of the New York Heart Association (NYHA). CAD was defined as documented coronary stenosis by angiography, history of myocardial infarction, previous coronary revascularization by angioplasty, stenting, or bypass surgery. Additionally, cardiac function was also evaluated according to parameters of pre-surgical echocardiographic findings.

### Definitions of echocardiographic findings

Two-dimensional echocardiography was performed at our institute using the Philips SONOS 7500 (Philips Healthcare, Philips International B.V, Amsterdam, The Netherlands). The following numeric parameters were assessed and recorded: left ventricular ejection fraction (LVEF), left ventricular end-diastolic diameter (LVEDD), and left ventricular end-systolic diameter. Nominal findings included the presence of chronic atrial fibrillation, moderate or greater degree impairment of the left ventricle (LVEF < 40%), moderate or greater left ventricle dilatation (LVEDD > 55 mm), moderate or greater left ventricle diastolic dysfunction, moderate or greater pulmonary hypertension, moderate or greater pericardial effusion, and moderate or greater valvular insufficiency and/or stenosis affecting the aortic, mitral, pulmonary, and tricuspid valves. The presence of each of the ten above-mentioned abnormal echocardiographic findings was given a score of one point; these were summed to create an echocardiographic score.

### Outcome measures

Occurrence of dialysis access dysfunction or occlusion requiring intervention was defined as a target access event (TAE). Major adverse patient events (MAE) included nonfatal myocardial infarction (MI), symptomatic CAD requiring coronary intervention, progression of CHF requiring hospitalization, nonfatal stroke or transient ischemic attack, worsening of limb ischemia requiring intervention, and major systemic infections requiring hospitalization based on medical records. We evaluated differences between chronic CVC and AVA patients in a number of outcomes, including: MAE-free survival (primary outcome), TAE, a composite end point comprising the incidence of death, MI, CAD, CHF, stroke, critical limb ischemia, major systemic infection, and finally, clinically and echocardiographically cardiac changes.

### Statistical analysis

An independent party with an academic affiliation who had access to the primary data served as the analyst for the investigators. Continuous variables were expressed as median and interquartile range (IQR; 25% and 75% quartiles). Categorical variables were expressed as frequency and percentage. Survivals were expressed by Kaplan-Meier curves and compared by Log-rank test. Univariate analyses of continuous variables were conducted by non-parametric Mann-Whitney U test, while those of categorical variables were conducted by Pearson’s Chi-square test (or Fisher exact test when expected count is <5). Predictors of survival were analyzed by multivariate Cox regression models. Comparisons of paired before-after clinical or echocardiographic parameters were conducted by Wilcoxon Signed Ranks test. Statistical analysis was completed using IBM SPSS Statistics software version 20 (IBM Corp. Armonk, NY). P≤.05 was considered statistically significant.

## Results

Between January 2007 and November 2008, a total of 121 consecutive patients with heart disease and end-stage renal disease underwent new haemodialysis access creations at our center. After excluding 18 patients for reasons described above (two had renal transplantation, 8 changed access type, and 8 switched to peritoneal dialysis), clinical records of 103 patients were available for this study, including 65 men and 38 women with the median age of 70.0 years (IQR 17.0, 63.0–80.0). Table 1 summarizes the clinical characteristics of patients of the two different types of access, i.e., CVC (n = 43; 41.7%), AVA (n = 60; 58.3%, including 47 AVF and 13 AVG patients). Most baseline characteristics and preoperatively echocardiographic parameters were identical between groups, with the exception of significantly older age among CVC patients than in AVA patients (median 78.0, IQR 14,0 year-old vs. 67.5, IQR 16.0 year-old, respectively, p = 0.009). Regardless of possible interventions, patients continued using the functional access for chronic haemodialysis. The median follow-up time from access creation was 27.6 months (IQR 34.7, 10.9~45.6).

**Table 1:** Clinical characteristics and echocardiographic parameters^a^.

	Accesses groups		
Characteristics ^b^	CVC	AVA	p value ^c^
Patient Number	43	60	
Age, yrs	78.0 (IQR 14.0, 67.0~81.0)	67.5 (IQR 16.0, 61.0~77.3)	0.009*
Gender (M/F)	28/15	37/23	0.720
BMI, kg/m2	22.4 (IQR 6.5, 20.2~26.7)	23.9 (IQR 6.5, 20.5~27.0)	0.511
BSA, m2	1.6 (IQR 0.2,1.5~1.7)	1.7 (IQR 0.3, 1.5~1.8)	0.768
Comobidities, n (%)			
Diabetes	24 (55.8%)	39 (65.0%)	0.360
Hypertension	40 (93.0%)	56 (93.3%)	0.687
Hyperlipidemia	10 (23.3%)	25 (41.7%)	0.056
Smoking	10 (23.3%)	16 (26.7%)	0.781
Cerebral vascular disease	15 (34.9%)	17 (28.3%)	0.552
Lung disease	23 (53.5%)	32 (53.3%)	0.814
Coronary artery disease	28 (65.1%)	38 (63.3%)	0.852
Peripheral artery disease	9 (20.9%)	8 (13.3%)	0.306
ADL dependence	12 (27.9%)	9 (15.0%)	0.109
Echocardiographic parameters			
Number	40	55	
LVEDD, mm	54.0 (IQR 7.0, 49.0~56.0)	52.5 (IQR 11.0, 46.0 ~56.0)	0.745
LVEF, %	53.0% (IQR 26.0%, 40.0~66.0%)	57.5% (IQR 18.0%, 47.8~65.3%)	0.124
LV dilatation, n (%)	13 (32.5%)	20 (36.4%)	0.696
LV systolic impairment, n (%)	11 (27.5%)	6 (10.9%)	0.037*
LV diastolic dysfunction, n (%)	20 (50.0%)	30 (54.5%)	0.661
Pulmonary hypertension, n (%)	19 (47.5%)	33 (60.0%)	0.227
Pericardial effusion, n (%)	13 (32.5%)	14 (25.5%)	0.452
Arrhythmia, n (%)	4 (10.0%)	3 (5.5%)	0.450
Aortic insufficiency, n (%)	7 (17.5%)	9 (16.4%)	0.884
Mitral insufficiency, n (%)	25 (62.5%)	24 (43.6%)	0.069
Pulmonary insufficiency, n (%)	4 (10.0%)	3 (5.5%)	0.450
Tricuspid insufficiency, n (%)	17 (42.5%)	19 (34.5%)	0.430

^a^ Preoperative echocardiographic data were available in 40/43 and 55/60 patients in the CVC and AVA groups, respectively. ADL, activity of daily living; AVA, arteriovenous access; BMI, body mass index; BSA, body surface area; CVC, tunneled cuffed double-lumen central venous catheter; LV, left ventricle; LVEDD, left ventricle end-diastolic diameter; LVEF, left ventricle ejection fraction.

^b^ Continuous variables are presented as median (interquartile range, percentile 25% ~ 75%); categorical variables are presented as number (percentage).

^c^ Comparisons of continuous variables were measured by non-parametric (independent samples Mann-Whitney U) tests, and those of categorical variables by Pearson's Chi-square test (or Fisher exact test when expected count is below 5).

* p < 0.05 signifies difference.

Of the 103 patients evaluated, 49 died during follow-up. Overall patient survival rates at 1, 2, and 5 years were 82.8%, 71.1%, and 30.6%, respectively (data in S1 Dataset). When clinical outcomes were compared (Table 2), CVC patients lived short as compared to AVA patients (31.6, 95% CI 24.5~38.7 months vs. 48.5, 95% CI 41.3~55.6, respectively, p = 0.010). Nevertheless, other outcomes and adverse event rates were similar between groups. Forty patients experienced TAEs that required one or more interventions to salvage the access, including 19 out of 43 CVC patients (44.2%) and 21 out of 60 AVA patients (35.0%). The elapsed times to first TAE were similar between groups.

**Table 2:** Clinical outcomes according to access types^a^.

	Groups		
Variable	CVC (n = 43)	AVA (n = 60)	p value ^b^
All-cause death, n (%)	24 (55.8%)	25 (41.7%)	
Survival time (months), mean (95% CI)	31.6 (24.5~38.7)	48.5 (41.3~55.6)	0.010*
Any TAE, n (%)	19 (44.2%)	21 (35.0%)	
Time to first TAE (months), mean (95% CI)	27.7 (18.2~37.2)	39.1 (31.4~46.7)	0.162
Any MAE, n (%)	31 (72.1%)	52 (86.7%)	
Time to first MAE (months), mean (95% CI)	17.1 (10.3~24.0)	12.9 (8.5~17.4)	0.290
Cerebral vascular event, n(%)	2 (4.7%)	7 (11.7%)	0.298
Coronary event, n (%)	9 (20.9%)	19 (31.7%)	0.227
CHF event, n (%)	14 (32.6%)	19 (31.7%)	0.924
Critical limb ischemia, n (%)	2 (4.7%)	3 (5.0%)	1.000
Major infection, n (%)	22 (51.2%)	28 (46.7%)	0.653

^a^ AVA, arteriovenous access; CI, confidence interval; CVC, tunneled cuffed double-lumen central venous catheter; CHF, congestive heart failure; MAE, major adverse event; TAE, target access event. Categorical variables are presented as number (percentage). Time variables are presented as mean and 95% confidence interval.

^b^ Comparisons of survival time (to first event) were measured by Log-rank tests; events were measured by Pearson's Chi-square test (or Fisher exact test when the expected count is below 5).

* p < 0.05 signifies difference.

**Fig 1:**
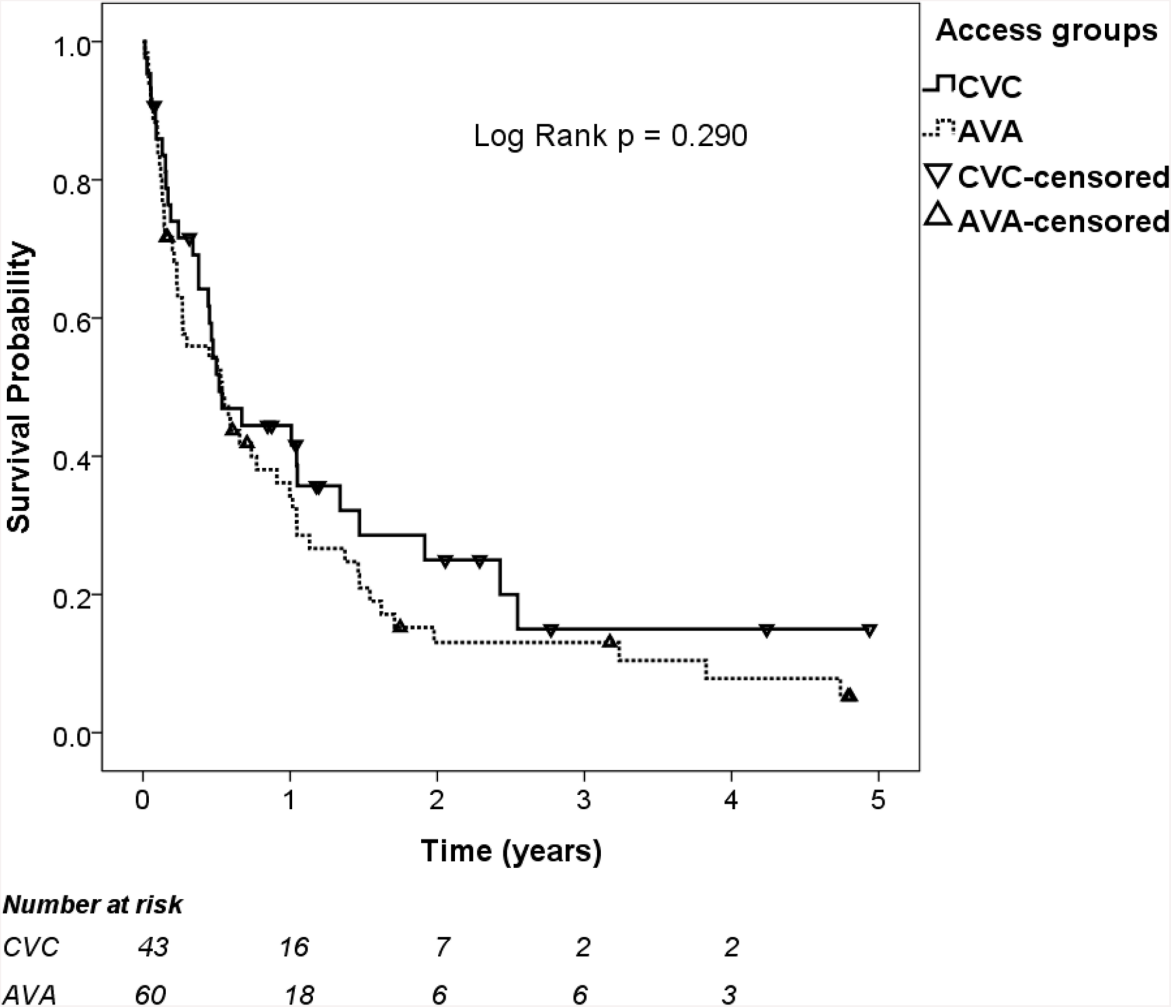
Major adverse event-free survivals are not significantly different between patient groups. AVA, arteriovenous access; CVC, tunneled cuffed double-lumen central venous catheter.

The end-point of no MAE nor death at 1, 2, and 5 years was reached by 39.7%, 18.9%, and 7.7% of patients, respectively. As depicted in Fig 1, MAE-free survivals were not significantly different between patient groups. Table 2 also lists the incidences of various MAEs during follow-up that no significant differences between groups were observed in any of these.

During follow-up, more patients in the AVA group presented with a worsening of their NYHA classes when compared to CVC patients (35 of 57 versus 10 of 42, respectively; Table 3). Echocardiographic scans were available both before and 3 months after surgery during follow up in 43 patients (17 in the CVC group, and 26 in the AVA group) that allowed before-after paired comparison of abnormal echocardiographic findings. More patients had pulmonary hypertension as compared to their each preoperative finding in both groups. However, mitral insufficiency and pulmonary insufficiency increased in AVA group only (Table 4). The counted-up echocardiographic scores increased in AVA group but remained relatively unchanged in the CVC group.

**Table 3:** Preoperative parameters and their associations with worsening of NYHA functional class.

	Worsening of NYHA class ^b^			
Variable ^a^	Yes (n = 45)	No (n = 54)	p value ^c^	OR (95%CI)
Groups				
CVC	10 (23.8%)	32 (76.2%)	<0.001*	1 (reference)
AVA	35 (61.4%)	22 (38.6%)		5.1 (2.1~12.4)
Age, yrs	70.0 (IQR 19.0, 61.0~80.0)	69.5 (IQR 22.0, 57.3 ~79.0)	0.323	
Gender (M/F)	32/13	31/23	0.158	
BMI, kg/m2	23.9 (IQR 5.9, 20.4~26.3)	22.0 (IQR 6.8, 20.2~27.0)	0.616	
BSA, m2	1.7 (IQR 0.2, 1.5~1.7)	1.6 (IQR 0.3, 1.5~1.8)	0.968	
Diabetes	28 (45.2%)	34 (54.8%)	0.940	
Hypertension	42 (44.7%)	52 (55.3%)	0.503	
Hyperlipidemia	20 (58.8%)	14 (41.2%)	0.053	
Smoking	13 (50.0%)	13 (50.0%)	0.588	
Cerebral vascular disease	15 (48.4%)	16 (51.6%)	0.692	
Lung disease	24 (44.4%)	30 (55.6%)	0.825	
Coronary artery disease	34 (52.3%)	31 (47.7%)	0.058	
Peripheral artery disease	7 (41.2%)	10 (58.8%)	0.224	
ADL dependence	9 (45.0%)	11 (55.0%)	0.964	

^a^ ADL, activities of daily living; AVA, arteriovenous access; BMI, body mass index; BSA, body surface area. CVC, tunneled cuffed double-lumen central venous catheter; OR, Odds ratio.

^b^ Continuous variables are presented as median (interquartile range, percentile 25% ~ 75%). categorical variables are presented as number (percentage).

^c^ Comparisons of continuous variables were measured by non-parametric tests (Mann-Whitney test), and those of categorical variables by Pearson's Chi-square test (or Fisher exact test when the expected count is below 5).

* p < 0.05 signifies difference.

**Table 4:** Preoperative-and-postoperatively pairwise comparison of echocardiographic abnormalities.

	CVC (N = 17)					AVA (N = 26) ^a^				
Parameter	Ranks					Ranks				
	Negative	Positive	Ties	Z value	p value	Negative	Positive	Ties	Z value	p value
LV dilatation	5	2	10	-1.13	0.257	4	2	20	-0.82	0.414
LV systolic impairment	2	3	12	+0.45	0.655	1	5	20	+1.63	0.102
LV diastolic dysfunction	2	5	10	+1.13	0.257	9	3	14	-1.73	0.083
Pulmonary hypertension	0	5	12	+2.25	0.025*	0	7	19	+2.65	0.008*
Arrhythmia	0	0	17	0.00	1.000	1	2	23	+0.58	0.564
Pericardial effusion	4	2	11	-0.82	0.414	5	2	19	-1.13	0.257
Aortic insufficiency	1	1	15	0.00	1.000	1	6	19	+1.90	0.059
Mitral insufficiency	5	1	11	-1.63	0.102	0	14	12	+3.74	<0.001*
Pulmonary insufficiency	1	3	13	+1.00	0.317	0	9	17	+3.00	0.003*
Tricuspid insufficiency	1	4	12	+1.34	0.180	0	3	23	+1.73	0.083
Echocardiographic score ^b^	6	5	6	+0.41	0.680	0	19	7	+3.95	< 0.001*

Comparison of each before-after pair was done by Wilcoxon signed ranks test.

* p < 0.05 signifies difference.

^a^ Forty three patients had echocardiographic scans before and after surgery, including 17 in the CVC group, and 26 in the AVA group.

^b^ Echocardiographic score was defined as a sum-up of the above 10 findings (see text). AVA, arteriovenous access; CVC, tunneled cuff double-lumen central venous catheter.

Subgroup regression analysis abstracted 60 AVA patients. Echocardiographic systolic impairment, pulmonary hypertension, and arrhythmia signified inferior MAE-free survival, while other cardiac parameters were unrelated (Table 5).

**Table 5:** Of AVA group, multivariate Cox regression analysis for MAE-free survival by preoperative echocardiographic parameters.

	Multivariate analysis	
Variable	Hazard Ratio (95%C.I.)	p value
Echocardiographic parameters		
LV dilatation (LVEDD > 55 mm)	0.77 (0.35~1.68)	0.507
LV systolic impairment (EF < 40%)	3.66 (1.13~11.79)	0.030*
LV diastolic dysfunction	1.23 (0.57~2.67)	0.595
Pulmonary hypertension	4.29 (1.98~9.28)	<0.001*
Pericardial effusion	2.05 (0.84~5.01)	0.117
Arrhythmia	6.11 (1.40~26.63)	0.016*
Aortic insufficiency	0.87 (0.36~2.08)	0.754
Mitral insufficiency	0.67 (0.36~1.25)	0.210
Pulmonary insufficiency	2.04 (0.55~7.48)	0.284
Tricuspid insufficiency	0.80 (0.39~1.62)	0.529

Multivariate model includes 10 categorically echocardiographic variables.

* p < 0.05 signifies difference.

AVA, arteriovenous access; CI, confidence interval; EF, ejection fraction; LVEDD, left ventricle end diastolic diameter.

## Discussion

In this retrospectively comparative cohort study, the MAE-free survival rates were similar between AVA and CVC groups of patients with symptomatic heart disease and uremia. A total of 49 patients died during follow-up, resulting in a 5-year survival rate of 30.6%. While the prognosis was poor, it is not unexpected given the complex co-morbidities and high rate of adverse events in this population.

Numerous patients initiate and maintain dialysis via a catheter. In the dialysis outcomes and practice patterns study (DOPPS), Ethier and colleagues found that at least 23% of haemodialysis patients used a catheter in the UK, Belgium, Sweden, Canada and the US at study entry in 2005–2007.[4] Despite the higher risk associated with catheter use in the DOPPS haemodialysis population as a whole, our study depicted a non-inferior outcome of 41.7% CVC use in patients with both symptomatic heart disease and ESRD requiring maintained haemodialysis.

Bias was present in the selection of access type for patients. Patients in the AVA group were younger and lived longer (median age of 67.5, mean survival time of 48.5 months) than those in CVC group (median age of 78.0, mean survival time of 31.6 months). Nevertheless, the difference in survival is less pronounced when comparing the life expectancies of people in Taiwan in 2007 at 67 and 77 years of age (17.3 and 10.8 years, respectively).[17] This study did not find a benefit of using AVA among patients with both heart disease and ESRD. When heart disease patients experience ESRD, it appears as though all common access types provided dialysis adequacy and similar MAE-free outcomes. With regard to rates of adverse events, the median follow-up time of 27.6 months could be another bias since patients could undergo more complications if they could be followed-up for a longer duration. Besides, patients admitted to the hospital with acute decompensated CHF could be related to, but not be equivalently translated into, their chronic heart failure.[18] This probably explains that there was no statistically difference in CHF events between groups (Table 2) despite the finding that AVA group had worsening of NYHA class over time (Table 3).

Since AV-type access represents a non-physiologic anomaly, [19] it might contribute to excess cardiac load and cardiovascular mortality among haemodialysis patients. Patients with AV type access in this study experienced deteriorating heart function in terms of both clinical NYHA class and echocardiographic parameters, meanwhile patients with CVC did not. Although the hemodynamic effects of a left-to-right shunt have been described, [20,21] a direct shunting effect on the heart was not identified in our echocardiographic findings.

Pulmonary hypertension has been detected in 40–50% of haemodialysis patients.[22] We found that when AV type access was created, 60% (33/55) of patients with heart disease already had moderate or worse pulmonary hypertension. Additionally, pulmonary hypertension was significantly related to time to first MAE (hazard ratio 4.29, 95% CI 1.98~9.28). Physicians had previously also argued that patients with LVEF below 40% are clinically inappropriate for an AVF.[19–22] In our analyses, moderate or worse impairment of LVEF (below 40%) was also significantly related to time to first MAE (hazard ratio 3.66, 95% CI 1.13~11.79).

While clinical guidelines for vascular access recommend avoiding CVC if possible, our findings reappraise the notion that an AVF may not be the optimal approach for each individual.[1] Indeed, we found that the AV type access contributed to a worsening of heart function while CVC provided non-inferior MAE-free outcomes in patients with both heart failure and ESRD.

Limitations of this study include a retrospective design, imperfect matching of patient groups on baseline characteristics contributing to a selection bias, and a limited life expectancy in this specific cohort. These limitations could decrease confidence in our findings. Additionally, no relations of flow and cardiac outcome could be explored due to lacking of routine surveillance of AVA flow in our patients. Moreover, lack of information regarding the presence of pacemaker or history of previous subclavian venous catheters also prevented us from elaborating the importance of central venous stenosis.

In summary, even though CVC patients with symptomatic heart disease and ESRD were older, they presented with non-inferior TAE-free access patency and MAE-free survival in comparison to those in the AVA group; AV type access could deteriorate heart failure; Pulmonary hypertension, systolic impairment, and arrhythmia signified inferior MAE-free outcome in those with an AV type access. Accordingly, patients with symptomatic heart disease are not ideal candidates for AV type access creation. Future well-matched studies are necessary to reappraise the influence of access type in patients with heart and kidney failure.

## Supporting Information

S1 Dataset Delinked data of 103 cases in this study. Identifying information was removed prior to analysis to protect patient privacy and anonymity.
(CSV)
